# Impact of perinatal administration of probiotics on immune cell composition in neonatal mice

**DOI:** 10.1038/s41390-024-03029-2

**Published:** 2024-01-26

**Authors:** Jessica Rühle, Julian Schwarz, Stefanie Dietz, Xenia Rückle, Ulrich Schoppmeier, Trim Lajqi, Christian F. Poets, Christian Gille, Natascha Köstlin-Gille

**Affiliations:** 1 Department of Neonatology, Tuebingen University Children’s Hospital, Tuebingen, Germany; 2 Department of Neonatology, Heidelberg University Children’s Hospital, Heidelberg, Germany; 3 Institute for Medical Microbiology and Hygiene, University Hospital Tuebingen, Tuebingen, Germany

## Abstract

**Background:**

Newborns and especially preterm infants are much more susceptible to infections than adults. The pathogens causing infections in newborns are often detectable in the intestinal flora of affected children even before disease onset. Therefore, it seems reasonable to prevent dysbiosis in newborns and preterm infants. An approach followed in many neonatal intensive care units (NICUs) is to prevent infections in preterm infants with probiotics however their mechanisms of action of probiotics are incompletely understood. Here, we investigated the effect of perinatal probiotic exposure on immune cells in newborn mice.

**Methods:**

Pregnant mice were orally treated with a combination of *Lactobacillus acidophilus* and *Bifidobacterium bifidum* (Infloran®) from mid-pregnancy until the offspring were harvested. Immune cell composition in organs of the offspring were analyzed by flow cytometry.

**Results:**

Perinatal probiotic exposure had profound effects on immune cell composition in the intestine, liver and lungs of newborn mice with reduction of myeloid and B cells and induction of T cells in the probiotic treated animals’ organs at weaning. Furthermore, probiotic exposure had an effect on T cell development in the thymus.

**Conclusion:**

Our results contribute to a better understanding of the interaction of probiotics with the developing immune system.

**Impact:**

- probiotics have profound effects on immune cell composition in intestines, livers and lungs of newborn mice.
- probiotics modulate T cell development in thymus of newborn mice.
- effects of probiotics on neonatal immune cells are particularly relevant in transition phases of the microbiome.
- our results contribute to a better understanding of the mechanisms of action of probiotics in newborns.

## Introduction

Probiotics are defined as live microorganisms which, when administered in adequate amounts, confer a health benefit to the host.^1^ They have beneficial effects on a wide range of diseases such as inflammatory bowel disease (IBD),^2^ allergies,^3^ obesity,^4^ autoimmune diseases,^5^ and many others. In preterm infants, prophylactic efficacy against necrotizing enterocolitis (NEC), late-onset sepsis, feeding-intolerance and all-cause mortality has been described^6–9^ leading to the routine use of probiotics in many neonatal intensive care units (NICUs) worldwide.^10–12^ The presumed mechanisms of action of probiotics are diverse. In addition to influencing the bacterial composition of the microbiome directly, for example by production of antimicrobial proteins or competitive adhesion to the intestinal mucosa,^13^ probiotics can enhance barrier functions of the intestinal mucosa and modulate intestinal immunity.^10,14^

In the newborn, microbiome and immune system develop in parallel and influence each other. While during pregnancy the main task of the fetal immune system is to protect itself from rejection by the maternal immune system, immediately after birth, it must “learn” to fight infections and at the same time tolerate the colonization of body surfaces (skin, airways, gastrointestinal tract) by trillions of bacteria to ensure the establishment of a diverse, robust microbiota. Alterations of the microbiome can lead to far-reaching changes in the development of the immune system and thus pre-program the newborn’s susceptibility to later inflammatory diseases. For example, early antibiotic exposure has not only been associated with a wide variety of diseases such as allergies, IBD and asthma,^15–17^ but also seems to influence immune cell development in the thymus and immune responses in the context of vaccination in animal models.^18,19^ Therefore, modulation of the microbiome during the so called “window of opportunity” is a very promising approach to direct immune programming.^20^

In this study, we investigated the influence of very early probiotic exposure on the major immune cell populations in the gastrointestinal tract and lungs and on thymic T cell development in newborn and growing mice.

## Methods

### Mice

C57BL/6J mice were obtained from Charles River (Sulzfeld, Germany). All animals were maintained under pathogen-free conditions in the research animal facility of Tuebingen University, Tuebingen, Germany (K10/18G). All experimental animal procedures were conducted according to German federal and state regulations.

C57BL/6J mice were mated and treated with either probiotics (1 × 10^9^ CFU *Lactobacillus acidophilus* and *Bifidobacterium bifidum* (Infloran®, Laboratorio Farmaceutico SIT, Adelaide, Australia) once per day via button probe) or water from day 10 of gestation. Probiotic administration to the dams was carried out until pups were harvested. This probiotic combination was chosen as it is routinely used in many NICUs worldwide for the prevention of NEC.^12,21–23^

### Tissue collection and single cell preparations

Newborn mice were sacrificed on postnatal days 3 (P3), 7 (P7), 14 (P14), and 21 (P21), and intestines, livers, lungs, thymi and spleens removed. Tissue from livers (whole liver), lungs (right lung lobe), thymi (whole thymus) and spleens (whole spleen) was homogenized using a 70 µm and 40 µm filter (Greiner bio-one, Frickenhausen, German) and a syringe plunger to obtain single cell suspensions.

Intestines were removed and stool samples collected from the colon. The stool samples were immediately frozen at −80 °C until further processing. Small intestines were washed with PBS and incubated in PBS with 5 mM EDTA and 10% FCS for 30 min to separate intestinal epithelial cells. Intestines were then homogenized using a scissor and incubated with 0.5 mg liberase (Roche, Basel, Switzerland) for up to one hour. Afterwards, lamina propria leukocytes were separated by Percoll density gradient centrifugation (cells were resuspended in 40% Percoll (Cytiva, Marlbourogh) and layered on 70% Percoll).

All single cell suspensions were then adjusted to 1–5 × 10^6^ cells/ml in PBS.

### Flow cytometry

For extracellular staining of mouse cells, freshly isolated cells were washed in FACS buffer and fluorescent-conjugated extracellular antibodies added. Antibodies were purchased from BD Biosciences (CD3 FITC (145-2C11), CD4 APC (RM4-5), CD4 FITC (RM4-5), CD8a APC-H7 (53-6.7), CD11b FITC (M1/70), CD19 PE (1D3), CD45 PerCp-Cy5.5 (30-F11), FSV510 Amcyan) or from Biolegend (CD11c BV421 (N418), F4/80 APC (BM8), Gr-1 PE-Cy7 (RB6-8C5), MHC II APC-Cy7 (M5/114.15.2) and Nkp46 PE-Cy7 (29A1.4). For immune cell quantification in intestines, livers, lungs, and spleens, cells were pre-gated to living CD45^+^ cells. Among these, immune cell subsets were defined as follows: T cells CD3^+^/CD19^−^, T Helper cells CD3^+^/CD4^+^, cytotoxic T cells CD3^+^/CD8^+^, B cells CD3^−^/CD19^+^, natural killer (NK) cells CD3^-^Nkp46^+^, myeloid cells CD11b^+^, neutrophilic cells CD11b^+^/Gr-1^+^, macrophages (liver, lung and spleen) CD11b^+^F4/80^+^, macrophages (intestine) CD11c^-^F4/80^+^, dendritic cells (DCs; liver, lung and spleen) CD11c^+^/MHC II^+^, DCs (intestine) CD11c^+^/F4/80^low^. Data acquisition was performed with a FACSCanto II and analyzed via FlowJo V10 (FlowJo, LLC, Ashland, Oregon).

### DNA extraction

DNA was extracted from stool samples using the ZymoBIOMICS^TM^ DNA Miniprep kit (ZymoResearch, Feiburg i. Br., Germany) according to manufacturer´s instructions. DNA concentration was measured using Varioskan^TM^ LUX Microplate Reader (Thermo Fisher Scientific, Waltham, MA).

### qPCR

The qPCR reaction was performed by QuantStudio 3 (Thermo Fisher Scientific, Waltham). All primers were pursued from MERCK (Darmstadt, Germany) with the following sequences: universal 16S forward AACTCAAAKGAATTGACGG and reverse CTCACRRCACGAGCTGAC; *Bacteroidetes* forward CRAACAGGATTAGATACCCT and reverse GGTAAGGTTCCTCGCGTAT; *Firmicutes* forward GGAGYATGTGGTTTAATTCGAAGCA and reverse AGCTGACGACAACCATGCAC; *Gammaproteobacteria* forward TCGTCAGCTCGTGTYGTGA and reverse CGTAAGGGCCATGATG; *Alphaproteobacteria* forward CIAGTGTAGAGGTGAAATT and reverse CCCCGTCAATTCCTTTGAGTT; *Actinobacteria* forward TACGGCCGCAAGGCTA and reverse TCRTCCCCACCTTCCTCCG; *Fusobacteria* forward AAGCGCGTCTAGGTGGTTATGT and reverse TGTAGTTCCGCTTACCTCTCCAG. The PCR reaction mix contained 2 µl of DNA (50 ng/µl), 0.5 µl forward primer [10 µM], 0.5 µl reverse primer [10 µM], 5 µl PowerUP^TM^ SYBR^TM^ Green Master Mix (Thermo Fisher Scientific, Waltham) and 2 µl nuclease­free water. The qPCR reaction was carried out according to the following protocol: 95 °C denaturation step for 10 min, followed by 45 cycles of denaturation at 95 °C for 15 s and annealing at 61.5 °C for 1 min. The melt curve analysis was performed by a dissociation step at 95 °C for 31 s and then for one sec at each 0.5 °C increment between 65 °C and 95 °C. Data collection was enabled at each increment of the melt curve. Analysis were performed using QuantStudio Design & Analysis Software (Version 2.6.0; Thermo Fisher Scientific, Waltham). Phylum composition was plotted as percentage of the total bacterial 16S load. In addition, the relative amount of each specific phyla was calculated in relation to the universal 16S load.

### Statistical analysis

Statistical analysis was done using GraphPad Prism 9.4.1 (GraphPad Software, La Jolla, CA). For comparison with two groups of unpaired and not normally distributed data the Mann–Whitney test was used and for comparison with two groups of unpaired and normally distributed date the unpaired *t* test was applied. A *p* value < 0.05 was considered statistically significant.

## Results

### Immune cell composition in untreated neonatal mice during the first three weeks

**Fig. 1:**
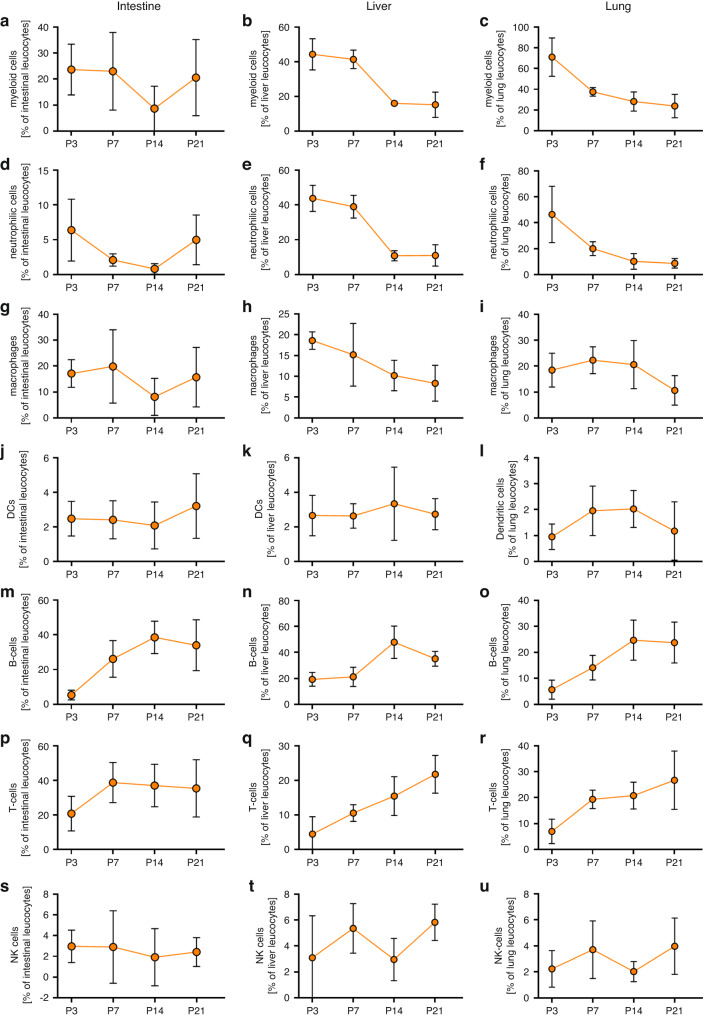
Immune cell composition in neonatal mice during the first three weeks of life. Organs of newborn C57BL/6J mice on postnatal day 3 (P3, *n* = 12), 7 (P7, *n* = 20), 14 (P14, *n* = 17), and 21 (P21, *n* = 13) and organs (intestines, livers, lungs) were analyzed by flow cytometry. **a**–**u** Line charts showing percentages of all myeloid cells (**a**–**c**), neutrophilic cells (**d**–**f**) macrophages (**g**–**i**), dendritic cells (DCs, **j**–**l**), B cells (**m**–**o**), T cells (**p**–**r**) and NK cells (**s**–**u**) from living CD45^+^ leukocytes in intestines (**a**, **d**, **g**, **j**, **m**, **p**, **s**), livers (**b**, **e**, **h**, **k**, **n**, **q**, **t**), and lungs (**c**, **f**, **i**, **l**, **o**, **r**, **u**) of newborn mice. Each symbol represents the mean of 12–20 individual samples and the standard deviation is indicated; *n* = 12–20.

First, we analyzed the main immune cell populations in intestine, liver and lung of newborn mice during the first three weeks after birth. In all organs investigated, myeloid cells made up the largest proportion of immune cells immediately after birth, with around 25% in intestine, 40% in liver and 70% in the lung (Fig. 1a–c). While neutrophilic cells were the most prominent myeloid cell population in liver and lung, macrophages made up the majority of myeloid cells in the intestine (Fig. 1d–i). Myeloid cells, especially neutrophilic cells dropped sharply from postnatal day 3 (P3) to postnatal day 21 (P21) in liver and lung (Fig. 1b, c, e, f). In intestine, there was a decrease of total myeloid cells, neutrophilic cells and macrophages by P14 and a rise again on P21 (Fig. 1a, d, g). B and T cells increased in all organs investigated from P3 to P21 (Fig. 1m–r). DC and NK cell numbers were largely stable at around 1–3% for DCs and 2–4% for NK cells (Fig. 1j–l and s–u). Supplementary Fig. 1 shows the gating strategy for these immune cell populations. In spleen, the course was similar to that in liver and lung, with a decrease in myeloid cells, neutrophilic cells and macrophages and an increase in B and T cells from P3 to P21. In spleen, there was also a slight increase in DCs and NK cells from P3 to P21 (Supplementary Fig. 2).

### Impact of perinatal probiotic exposure on gastrointestinal and lung immune cell composition in neonatal mice

**Fig. 2:**
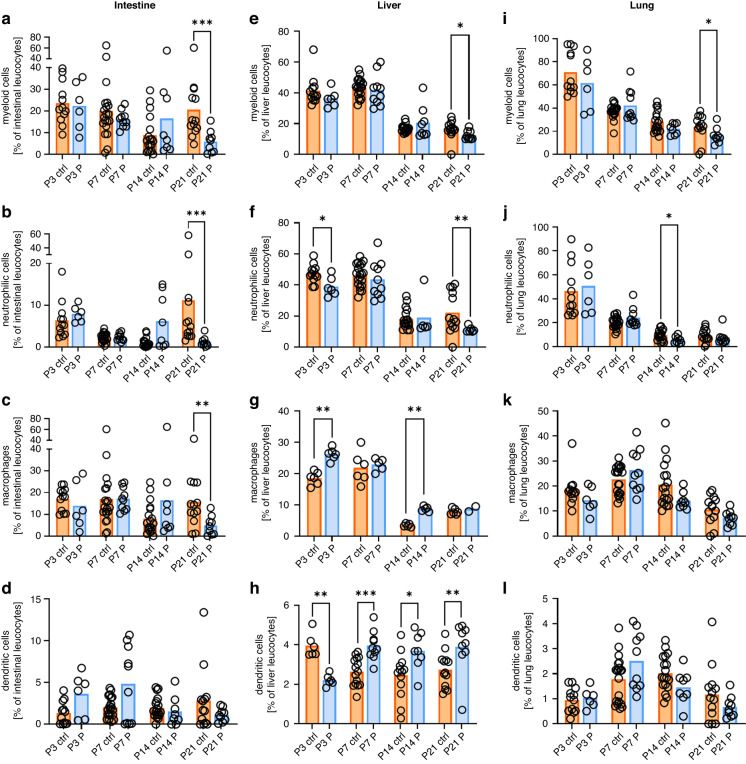
Myeloid cell populations in organs of newborn mice with and without probiotic treatment. Organs of newborn C57BL/6J mice on postnatal day 3 (P3, *n* = 12/6), 7 (P7, *n* = 20/10), 14 (P14, *n* = 17/8), and 21 (P21, *n* = 13/9) with or without probiotic supplementation of their dams with *Lactobacillus acidophilus* and *Bifidobacterium bifidum* were analyzed by flow cytometry. Scatter diagrams with bars showing percentages of total myeloid cells (**a**, **e**, **i**), neutrophilic cells (**b**, **f**, **j**) macrophages (**c**, **g**, **k**), and DCs (**d**, **h**, **l**) from living CD45^+^ leukocytes in intestines (**a**–**d**), livers (**e**–**h**), and lungs (**i**–**l**) of newborn mice with (blue bars) and without (orange bars) probiotic treatment. Each symbol represents an individual sample and the mean is indicated; *n* = 6–20, *p < 0.05; **p < 0.01; ***p < 0.001; Mann–Whitney test.

We then compared immune cell compositions in intestine, liver and lung of newborn mice with and without perinatal probiotic exposure. As the most prominent effect, we observed significantly decreased numbers of all myeloid cells (5.8% ± 5.1% vs. 20.6% ± 14.6%, *p* < 0.001, *n* = 9–13), neutrophilic cells (1.2% ± 1.2% vs. 11.2% ± 16.2%, *p* < 0.001, *n* = 9–13) and macrophages (4.9% ± 4.3% vs. 15.7% ± 11.5%, *p* < 0.01, *n* = 9–13) at P21 in the intestines of probiotic-treated animals (Fig. 2a–c). A similar but less pronounced effect was observed for myeloid cells and neutrophilic in livers (12.3% ± 2.9% vs. 16.2% ± 5.8%, *p* < 0.05, *n* = 9–13 for total myeloid cells and 11.3% ± 1.8% vs. 22.3% ± 13.6%, *p* < 0.01, *n* = 9–13 for neutrophilic) and lungs (16.2% ± 6.8% vs. 23.8% ± 11.3%, *p* < 0.05, *n* = 9–13 for total myeloid cells and 7.4% ± 5.9% vs. 9.3% ± 5.5%, *p* = 0.2, *n* = 9–13 for neutrophilic) (Fig. 2e, f,i and j). Macrophages were increased after probiotic treatment in livers at P3 and P14 (26.2% ± 1.9% vs. 18.6% ± 2.1%, *p* < 0.01, *n* = 6 at P3 and 8.8% ± 0.8% vs. 3.5% ± 0.5%, *p* < 0.01, *n* = 5, Fig. 2g) and DCs were decreased in livers at P3 (2.2% ± 0.3% vs. 3.9% ± 0.6% *p* < 0.01, *n* = 6) but increased at P7 (3.9% ± 0.7% vs. 2.6% ± 0.7%, *p* < 0.01, *n* = 10–15), P14 (3.7% ± 1.0% vs. 2.5% ± 1.1%, *p* < 0.001, *n* = 8–13) and P21 (3.9% ± 1.3% vs. 2.7% ± 0.9%, *p* < 0.01, *n* = 9–13) after probiotic treatment (Fig. 2h). No differences were found for lung macrophages and intestinal and lung DCs (Fig. 2d, k, l).

**Fig. 3:**
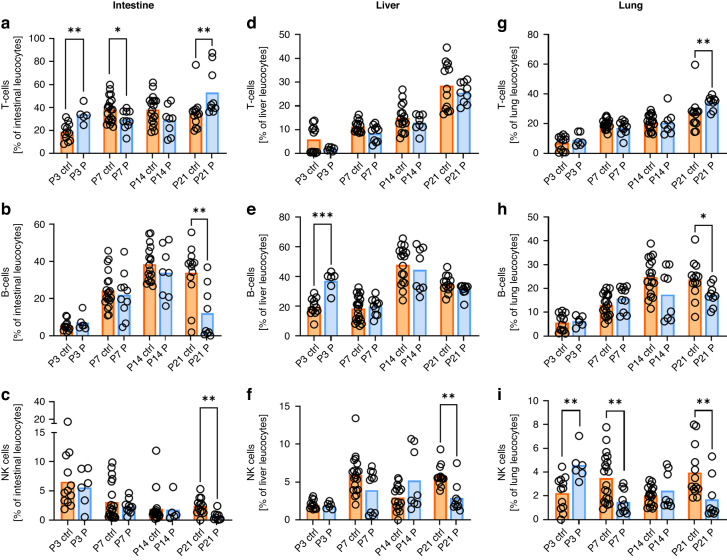
Lymphoid cell populations in organs of newborn mice with and without probiotic treatment. Organs of newborn C57BL/6J mice on postnatal day 3 (P3, *n* = 12/6), 7 (P7, *n* = 20/10), 14 (P14, *n* = 17/8), and 21 (P21, *n* = 13/9) with or without probiotic supplementation of their dams with *Lactobacillus acidophilus* and *Bifidobacterium bifidum* were analyzed by flow cytometry. Scatter diagrams with bars showing percentages of total T-cells (**a**, **e**, **i**), B-cells (**b**, **e**, **h**), NK-cells (**c**, **f**, **i**) from living CD45^+^ leukocytes in intestines (**a**–**c**), livers (**d**–**f**), and lungs (**g**–**i**) of newborn mice with (blue bars) and without (orange bars) probiotic treatment. Each symbol represents an individual sample and the mean is indicated; *n* = 6–20, *p < 0.05; **p < 0.01; Mann–Whitney test.

In lymphocytes, we found increased proportions of T cells and decreased proportions of B cells in intestines (53.1% ± 20.9% vs. 35.3% ± 14.3%, *p* < 0.01, *n* = 9–13 for T cells and 12.1% ± 14.1% vs. 34.0% ± 14.6%, *p* < 0.01, *n* = 9–13 for B-cells) and lungs at P21 (34.2% ± 4.2% vs. 26.7% ± 11.3%, *p* < 0.01, *n* = 9–13 for T cells and 17.2% ± 4.5% vs. 23.8% ± 7.9%, *p* < 0.05, *n* = 9–13 for B cells) after probiotic treatment (Fig. 3a, b, i, j). In livers, no differences were observed for T cells (Fig. 3d), while B cells were increased at P3 (37.1% ± 6.5% vs. 19.6% ± 5.7%, *p* < 0.001, *n* = 6–12) in probiotic treated animals (Fig. 3e). NK cells were decreased after probiotic treatment in all organs investigated at P21 (0.7% ± 0.8% vs. 2.4% ± 1.4%, *p* < 0.01, *n* = 9–13 for intestine, 2.9% ± 1.9% vs. 5.8% ± 1.4%, *p* < 0.01, *n* = 9–13 for livers and 1.7% ± 1.7% vs. 4.0% ± 2.2%, *p* < 0.01, *n* = 9–13 for lungs) (Fig. 3c, f, i).

### Microbiome composition in neonatal mice during the first three weeks with and without oral administration of probiotics

**Fig. 4:**
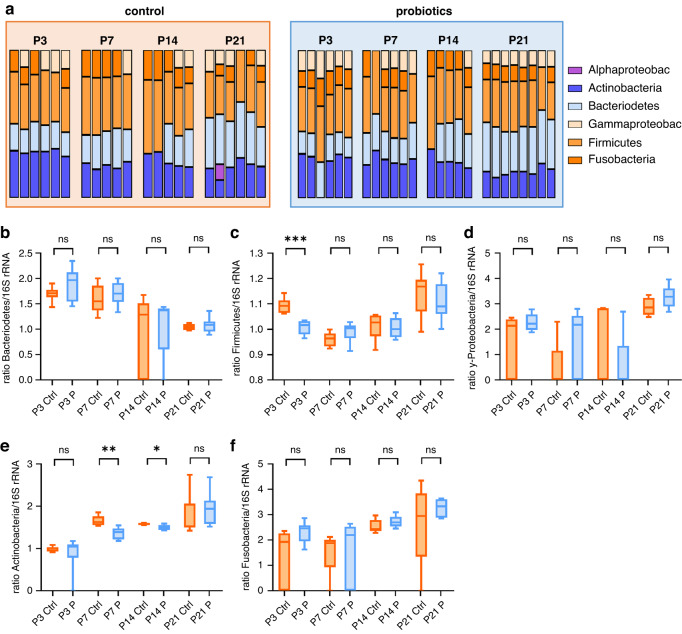
Microbiome composition in neonatal mice after probiotic treatment evaluated by qPCR. Stool samples (P3: *n* = 6, P7: *n* = 5–6, P14: *n* = 5, P21: *n* = 6–8) from newborn C57BL/6J mice with or without probiotic supplementation of their dams with *Lactobacillus acidophilus* and *Bifidobacterium bifidum* were collected and analyzed by qPCR for the six main phyla of the microbiome. **a** Bar graphs showing the relative abundance of the six phyla analyzed in stool samples from control animals and probiotic treated animals at the different timepoints. Box plots showing the abundance of *Bacteroidetes* (**b**), *Firmicutes* (**c**), *γ-Proteobacteria* (**d**), *Actinobacteria* (**e**), and *Fusobacteria* (**f**) relative to total 16S rRNA in control mice (orange box plots) and probiotic treated mice (blue box plots). *n* = 5–8, *p < 0.05, **p < 0.01, ***p < 0.001, unpaired *t* test and Mann–Whitney test.

We next examined the relative abundances of the six major phyla in feces of newborn mice by qPCR according to previous studies.^24,25^ In terms of the proportion of each phylum among the phyla analyzed, we found a decrease in *Actinobacteria* from around 30% to around 20% and an increase in *Bacteroidetes* from around 20% to 30% from P3 to P21, while proportions of *Firmicutes* (~35%), *Fusobacteria* (~11%) and *γ-Proteobacteria* (~7%) remained stable throughout in both, control animals and probiotic treated animals. *α-Proteobacteria* were detected in only one sample (Fig. 4a). Relative to the total amount of 16S rRNA, probiotic-treated animals had fewer *Firmicutes* at P3 (*p* < 0.001, *n* = 6) and fewer *Actinobacteria* at P7 and P14 (*p* < 0.05 and *p* < 0.01, *n* = 5–6, Fig. 4c, e); no differences were seen in the other phyla (Fig. 4b, d, f).

### Perinatal probiotic exposure leads to altered thymic T cell development

**Fig. 5:**
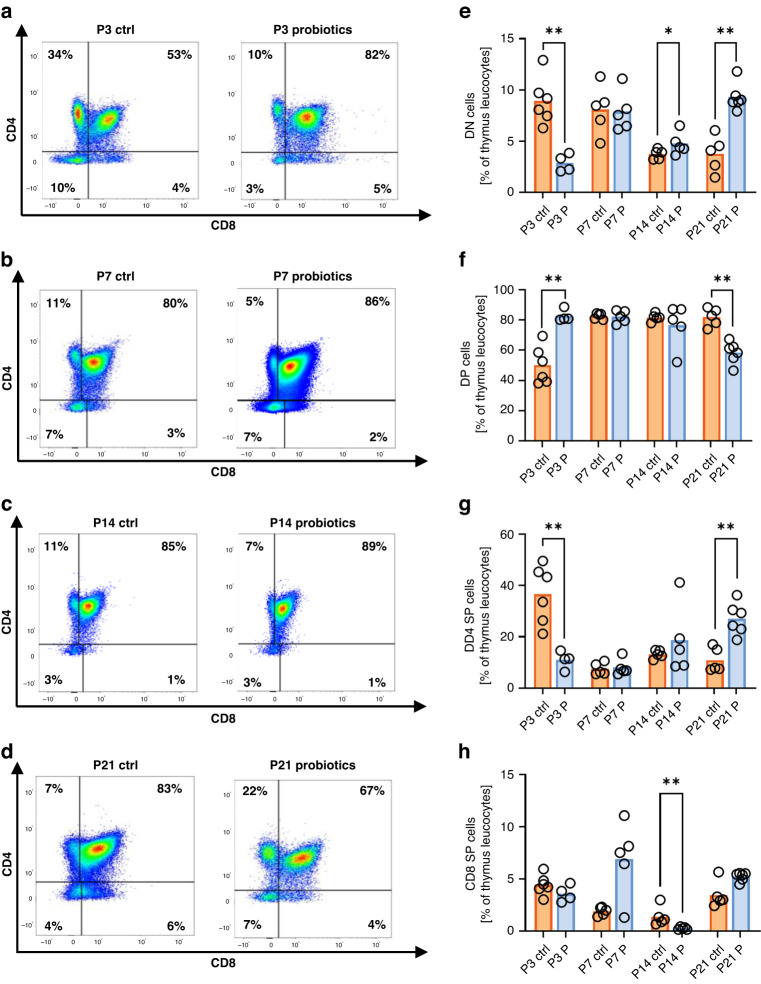
Thymic T cell maturation after neutrophil depletion in neonatal mice. Thymi of newborn C57BL/6J mice postnatal day 3 (P3, *n* = 6/4), 7 (P7, *n* = 5/5), 14 (P14, *n* = 5/5), and 21 (P21, *n* = 6/6) with or without probiotic supplementation of their dams with *Lactobacillus acidophilus* and *Bifidobacterium bifidum* were analyzed by flow cytometry. Density plots for CD8 vs. CD4 showing the populations of single positive (SP), double negative (DN) and double positive (DP) thymocytes in thymi of newborn WT mice with (probiotics) or without (ctrl) probiotic treatment at P3 (**a**) and P7 (**b**), P14 (**c**), and P21 (**d**). Cells were pre-gated on living lineage negative cells. Scatter diagrams with bars showing percentages of DN (**e**), DP (**f**), SP CD4^+^ thymocytes (**g**), and SP CD8^+^ thymocytes (**h**) in thymi of newborn mice with (blue bars) and without (orange bars) probiotic treatment. Each symbol represents an individual sample and the mean is indicated; *n* = 4–6, *p < 0.05; **p < 0.01; Mann–Whitney test.

Finally, we examined thymocyte populations in probiotic treated mice and control animals to determine whether early exposure to probiotics might already be influencing T cell development in the thymus. Thymocytes mature from double negative (DN) cells over double positive (DP) to single positive (SP) cells. We found decreased levels of DN (2.8% ± 0.9% vs. 8.9% ± 2.3%, *p* < 0.01, *n* = 4–6, Fig. 5a, e) and SP CD4^+^ thymocytes (10.9 ± 3.4% vs. 36.6% ± 1.3%, *p* < 0.01, *n* = 4–6, Fig. 5c, g) and increased levels of DP thymocytes after probiotic treatment at P3 (82.6% ± 4.0% vs. 50.0% ± 12.4%, *p* < 0.01, *n* = 4–6, Fig. 5b, f). While we observed hardly any differences at P7 and P14, P21 showed the opposite picture to P3 with increased levels of DN (9.3% ± 1.3% vs. 3.8% ± 1.8%, *p* < 0.01, *n* = 5–6, Fig. 5a, e) and SP CD4^+^ thymocytes (27.1 ± 6.2% vs. 10.9% ± 4.7%, *p* < 0.01, *n* = 5–6, Fig. 5c, g) and decreased levels of DP thymocytes in probiotic treated pups (58.4% ± 7.2% vs. 81.9% ± 5.9%, *p* < 0.05, *n* = 5–6, Fig. 5b, f).

## Discussion

In our in vivo study we investigated immune cell composition in intestine, liver and lung of newborn mice with and without perinatal exposure to *Lactobacillus acidophilus* and *Bifidobacterium bifidum*. We observed a pronounced effect of probiotic treatment on both, myeloid and lymphoid immune cells, especially immediately after birth and again at the time of weaning, suggesting that probiotics have immunological effects particularly during periods of major microbiome changes.

In untreated pups we showed that innate immune cell populations, especially neutrophilic cells and macrophages, sharply decreased after birth until the age of three weeks in all organs investigated, while adaptive immune cells increased. This is in line with a study in calves, where the number of myeloid cells in intestines decreased between birth and weaning, while T cells increased.^26^ It has also been shown in humans that the proportion of innate immune cells decreased during the first weeks of life, while cells of the adaptive immunity increased.^27^ This is probably because newborns are primarily dependent on innate immunity immediately after birth due to very limited antigen exposure in utero, whereas adaptive immune functions just begin to develop after antigen contact in the first weeks and months.^28^ However, it should be noted that not only the number of innate immune cells differs between newborns and adults, but also their functionality.^29–35^ Functional differences between immune cell populations in mice with or without probiotics were not considered in our study, but should be analyzed in further studies.

We found differences in immune cell composition between probiotic-treated pups and control animals, particularly early after birth (P3) and then again at the time of weaning (P21). This observation is noteworthy because both are times where major changes in the microbiome occur acutely (immediately after birth) or slowly (at weaning). Immediately after birth, the previously sterile intestine of the newborn is rapidly colonized with microorganisms; at the time of weaning, the change in food intake from breast milk to solid food also has a significant impact on the microbiome in terms of an increase in absolute quantity, diversity and metabolic activity.^36,37^ The observation that especially at these time points the probiotic supplementation has an impact on immune cell populations, whereas in between there are much more stable conditions, could indicate that the immunomodulatory effects by probiotics are particularly relevant in transition phases of the microbiome. However, further investigations are necessary to confirm our observations.

For innate immune cells, we observed an effect of probiotic supplementation in particular at the time of weaning with decreased numbers of myeloid cells, especially neutrophilic cells and macrophages, and decreased numbers of NK cells in probiotic-treated animals. Similar to our findings, a study on immune cell populations of newborn mice whose mothers were supplemented with *Lactobacillus casei* during lactation, also found decreased numbers of intestinal macrophages and DCs at day 12 after birth.^38^ This may lead to a reduction in phagocytotic activity and antigen presentation at the time of weaning which could be beneficial for the diversity of the developing microbiome in terms of protection against exaggerated immune reactions against the newly colonizing commensals.

In lymphoid cells, we observed increased levels of T cells after probiotic treatment at P3 and at the time of weaning. Similar observations were made by Moreno de le Blanc et al.^38^ while others found no increase in T cells after Lactobacillus supplementation.^39,40^ However, it is clear from the literature that various probiotic strains can induce regulatory T cells both in vitro and in vivo.^41–43^ Ekmekciu et al. also observed that, after 8 weeks of broad-spectrum antibiotic therapy, systemic and organ-specific populations of regulatory T cells and memory T cells could be restored by recolonization with probiotics.^44^ Unfortunately, we have no data on the presence of regulatory T cells in the organs of these pups. Conversely, we observed a decreased proportion of B cells at the time of weaning after probiotic supplementation. This is in contrast with the existing literature, suggesting that probiotics modulate gene expression associated with B cell activation, promote the development and maturation of B cells, enhance the activation and antigen-presentation ability of B cells and regulate their immunoglobulin secretion.^45^ However, the effect of probiotics on B cells seems to depend on the timing of application and it is perceivable that suppression of B cells and their antigen-presenting capacity at a time of upheaval in the microbiome (such as weaning) is necessary to create the conditions for a successful colonization.^46^ Similar to myeloid cells, NK cells were decreased under probiotic therapy at the time of weaning. As discussed for the myeloid cell populations, we hypothesize that probiotics reduce a nonspecific response by innate immune cells to enable adequate colonization of the mucosal surfaces.

We found very similar effects of probiotics on intestinal and lung immune cell populations. It is known that via the production of metabolites by microorganisms of the intestinal microbiome (for example small chain fatty acids), not only the local immune response is modulated, but also that at more distant sites such as the respiratory tract (the so called gut-lung axis).^47^ In addition, there also seems to be a direct migration of immune cells from the intestine to the lungs.^48^ Various studies showed that enteral administration of probiotics has a beneficial effect on respiratory diseases.^49–51^ The extent to which the immunological changes we observed play a role here should be the subject of further studies.

Comparable findings were observed in the liver too, albeit less pronounced, with immunological changes as in the intestine and lungs. Since the liver is the first organ in the circulation downstream of the intestine, metabolites from the intestine enter the liver directly. Conversely, the liver communicates with the intestine via the bile ducts. This bidirectional communication between intestine and liver leads to mutual influence of immunological processes.^52^ A difference from intestines and lungs was observed in DCs. Here, probiotic treatment resulted in a decreased expression at P3, and an increased expression at all other time points. Compared with secondary lymphoid tissue DCs, hepatic DCs were shown to be immature and less immunogenic, express lower levels of MHC class II and costimulatory molecules and are less effective in priming naïve allogeneic T cells.^53–55^ Induction of this more tolerogenic cell population by probiotics could contribute to increased tolerance to the developing microbiome.

From our data, it remains unclear how the effects of probiotic therapy on the newborn immune system are mediated. There is good data that the maternal microbiome already has an influence on the infants immunity.^56^ In addition, there are a number of in vitro studies that showed a direct effect of probiotics on adult and neonatal immune cells.^57–60^ Differences in the establishing microbiome between offspring with and without probiotic supplementation could also be responsible for the immunological differences. Which of these effects is particularly relevant should be the subject of further studies in order to obtain evidence as to when the optimal time to start probiotic treatment is.

We analyzed the microbiome in newborn and growing mice with and without probiotic therapy only at the phylum level. It is known that the composition of the microbiome is associated with the development of inflammatory diseases in child- and adulthood.^61–64^ We found only minor differences at the phylum level between mice with and without perinatal probiotic therapy. Looking at studies on probiotics in preterm and term neonates, a common finding is that there is enrichment of the administered strains in the microbiome.^9^ However, observations regarding larger colonization patterns vary widely. While some studies have demonstrated a significant effect on microbial composition after probiotic treatment,^65,66^ others found no effect.^67^ Interestingly, on a phylum level, we found *Actinobateria* (to which *Bifidobacterium bifidum* belongs) and *Firmicutes* (to which *Lactobacillus acidophilus* belongs) to be decreased rather than increased after probiotic supplementation. However, we did not perform more in-depth microbiome analyzes, so we cannot outline whether there was an enrichment of the applied strains in our experiments.

Finally, we also observed an influence of perinatal probiotic exposure on T cell development in the thymus. In particular, there was a shift in the ratio of DN and DP thymocytes at P3 and P21, a reduction of SP CD4^+^ T cells at P3 and an induction of SP CD4^+^ T cells at P21. Similar to the periphery, an influence of probiotics on thymic immune cell populations was observed early after birth (P3) and at the time of weaning (P21). An influence of probiotic therapy on T cell development in the thymus has already been shown by others.^68^ In addition, the microbiome influences the development of T cells in the thymus during early life.^18^ Interestingly, in contrast to the development of conventional T cells, innate-like T cells such as invariant Natural Killer T (iNKT) cells and Mucosal-associated Invariant T (MAIT) cells are mostly developed from DP thymocytes.^69^ Induction of this population by probiotics immediately after birth suggests that at this time, nonspecific innate immune functions are supported, whereas at the time of weaning, when mature SP CD4^+^ T cells are induced, the then more developed adaptive immunity is strengthened.

In conclusion, here we showed that oral probiotics had an impact on peripheral immune cell composition and thymic T cell development. The results of our study contribute to a better understanding of the effect of probiotics on the neonatal immune system. However, it must be mentioned that no direct conclusions can be drawn from our results for clinical use of probiotics in newborns and preterm infants. A large number of studies investigated the effects of various probiotics on NEC, sepsis and mortality in preterm infants (recently reviewed in ref. ^7^). Overall, there were rather favorable effects, especially on NEC, but the effectiveness depended strongly on the probiotic strains used. For the strains investigated here, there are mainly retrospective data that analyzed the incidence of NEC before and after the introduction of a routine application on NICUs and found a protective effect.^12,22,23^ The extent to which the immunological changes we observed in our study contribute to this effect remains unclear. More detailed phenotypic and especially functional studies are needed to provide more precise information on the mechanisms of the protective effect of probiotics and their interaction with the immune system.

## Supplementary information

Suppl Figures

## Data Availability

The datasets generated during and/or analyzed during the current study are available from the corresponding author on reasonable requests.
