# Supplementary material for: Impact of perinatal administration of probiotics on immune cell composition in neonatal mice
Source: Pediatr Res. 2024 Jan 26;96(7):1645–54. doi: 10.1038/s41390-024-03029-2 (PMC11772233; doi:10.1038/s41390-024-03029-2)
Supplement: Supplementary file 1 — Suppl Figures [file 41390_2024_3029_MOESM1_ESM.pdf]

Supplementary Figure 1

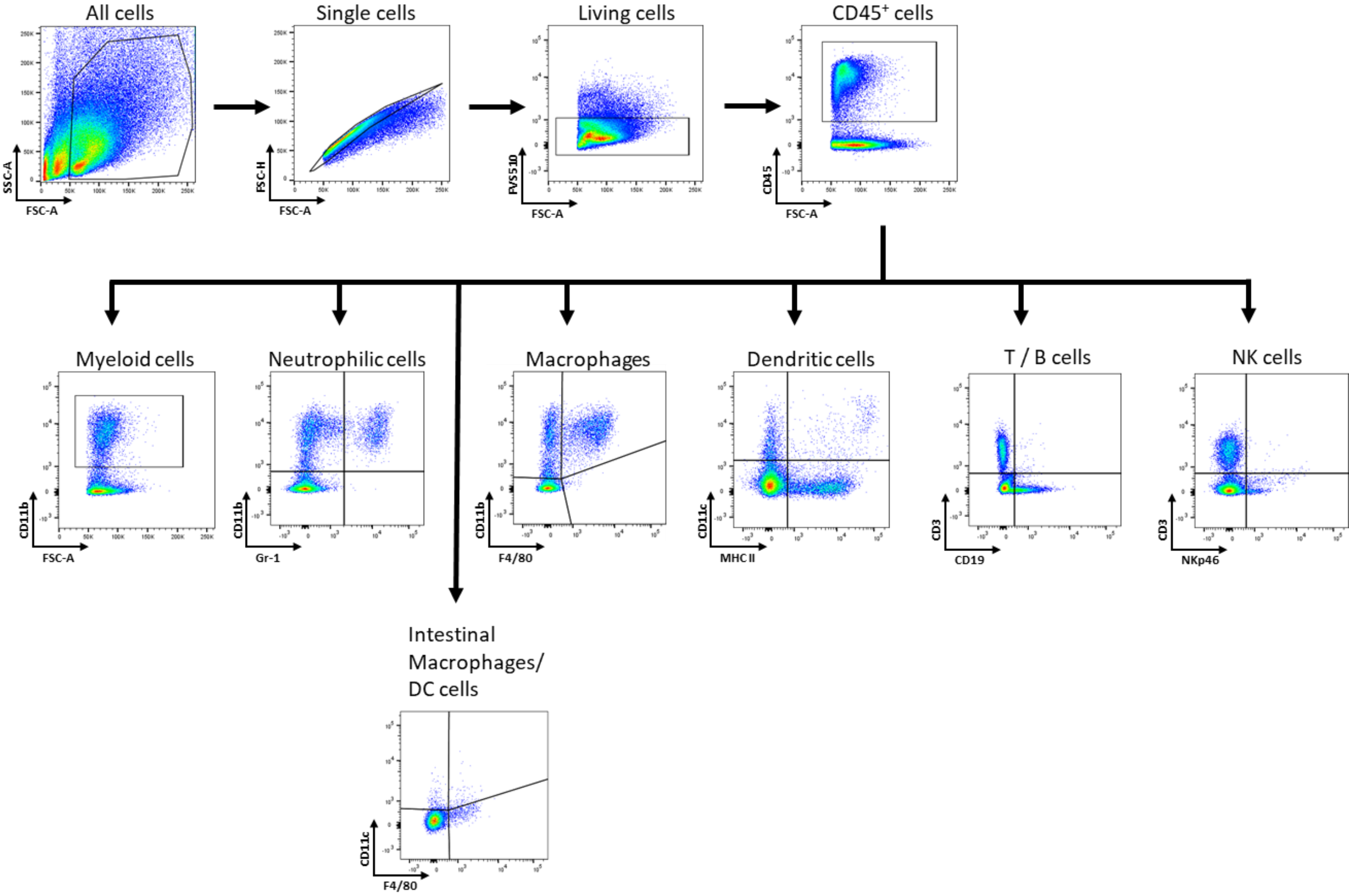

Supplementary Figure 1: Gating strategy for immune cell populations in lungs, livers, spleens and intestines.

Representative density plots show the gating strategy for immune cell populations in lungs, livers, spleens and intestines. Debris was excluded by gating on cells in SSC-A vs. FSC-A. Doublets and dead cells were then excluded and cells were pre-gated on $CD45^+$. Within $CD45^+$ leukocytes, immune cell subsets were defined as follows: myeloid cells $CD11b^+$, neutrophilic cells $CD11b^+$/$GR\text{-}1^+$, macrophages in livers, lungs, and spleens $CD11b^+$/$F4/80^+$, dendritic cells in livers, lungs and spleens $CD11b^+$/MHC II^+, macrophages in intestines $CD11c^-$$F4/80^+$, dendritic cells in intestines $CD11c^+$/$F4/80^{low}$, T cells $CD3^+$/$CD19^-$, B cells $CD3^-$/$CD19^+$ and NK cells $CD3^-$/$Nkp46^+$.

Supplementary Figure 2

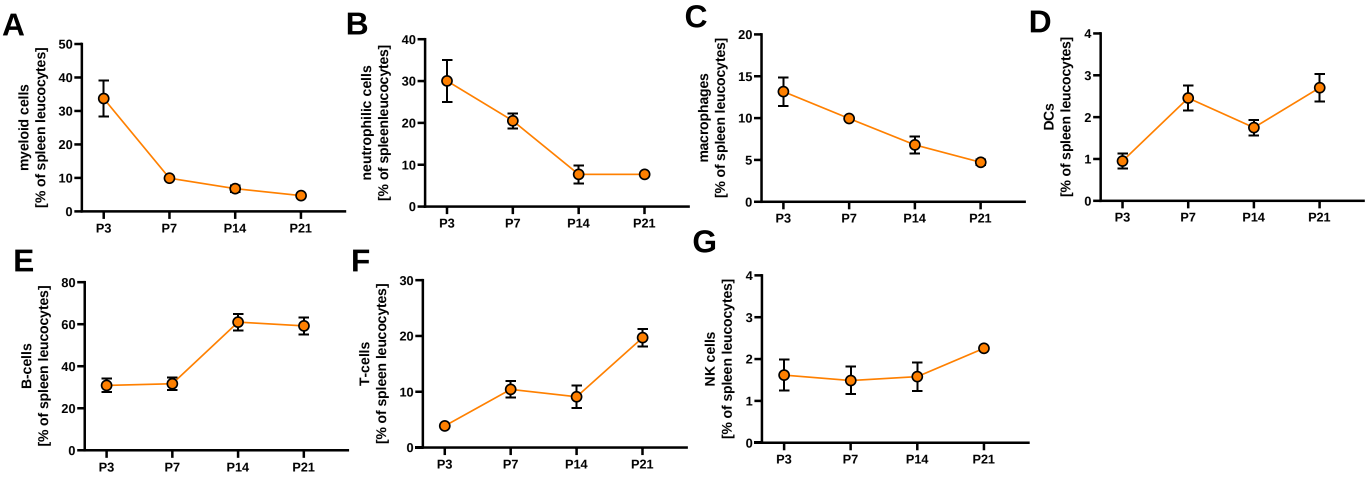

Supplementary Figure 2: Immune cell composition in neonatal mice during the first three weeks of life.

Newborn C57BL/6J mice were euthanized on postnatal day 3 (P3), 7 (P7), 14 (P14), and 21 (P21) and spleens were collected. Tissue was homogenized and filtered to obtain single cell suspensions. Cells were then analyzed by flow cytometry. (A-G) Line charts showing percentages of all myeloid cells (A), neutrophilic cells (B) macrophages (C), dendritic cells (DCs, D), B-cells (E), T-cells (F) and NK-cells (G) from CD45+ leucocytes in spleens of newborn mice. Each symbol represents the mean of 12-20 individual samples and the standard deviation is indicated; n=2-6.
